# Obstetric ultrasound use in low and middle income countries: a narrative review

**DOI:** 10.1186/s12978-018-0571-y

**Published:** 2018-07-20

**Authors:** Eunsoo Timothy Kim, Kavita Singh, Allisyn Moran, Deborah Armbruster, Naoko Kozuki

**Affiliations:** 1 Department of Maternal and Child Health, Gillings School of Global Public Health, University of North Carolina at Chapel Hill, 135 Dauer Dr, Chapel Hill, NC 27516 USA; 2 Carolina Population Center, University of North Carolina at Chapel Hill, Carolina Square, Suite 210, 123 West Franklin St, Chapel Hill, NC 27516 USA; 3 MEASURE Evaluation, Carolina Population Center, University of North Carolina at Chapel Hill, Carolina Square, Suite 330, 123 West Franklin St, Chapel Hill, NC 27516 USA; 4 US Agency for International Development, 1300 Pennsylvania Avenue, NW, Washington, DC 20523 USA; 5 International Rescue Committee, 1730 M St. NW Suite 505, Washington, DC 20036 USA; 6 Department of International Health, Johns Hopkins Bloomberg School of Public Health, 615 N. Wolfe St., Baltimore, MD 21205 USA

## Abstract

**Introduction:**

Although growing, evidence on the impact, access, utility, effectiveness, and cost-benefit of obstetric ultrasound in resource-constrained settings is still somewhat limited. Hence, questions around the purpose and the intended benefit as well as potential challenges across various domains must be carefully reviewed prior to implementation and scale-up of obstetric ultrasound technology in low-and middle-income countries (LMICs).

**Main Body:**

This narrative review discusses these issues for those trying to implement or scale-up ultrasound technology in LMICs. Issues addressed in this review include health personnel capacity, maintenance, cost, overuse and misuse of ultrasound, miscommunication between the providers and patients, patient diagnosis and care management, health outcomes, patient perceptions and concerns about fetal sex determination.

**Conclusion:**

As cost of obstetric ultrasound becomes more affordable in LMICs, it is essential to assess the benefits, trade-offs and potential drawbacks of large-scale implementation. Additionally, there is a need to more clearly identify the capabilities and the limitations of ultrasound, particularly within the context of limited training of providers, to ensure that the purpose for which an ultrasound is intended is actually feasible. We found evidence of obstetric uses of ultrasound improving patient management. However, there was evidence that ultrasound use is not associated with reducing maternal, perinatal or neonatal mortality. Patients in various studies reported to have both positive and negative perceptions and experiences related to ultrasound and lastly, illegal use of ultrasound for determining fetal sex was raised as a concern.

## Plain English summary

Prior to the implementation and scale-up of obstetric ultrasound technology in low- and middle-income countries, questions around the purpose, intended benefit, potential trade-offs and challenges must be carefully reviewed. This review paper focuses on issues around health personnel capacity, maintenance, cost, misuse and overuse, miscommunication between patients and providers, patient diagnosis and case management, health outcomes, perceptions and concerns about fetal sex determination.

We conclude that it is essential to assess the benefits, trade-offs and potential drawbacks of large-scale implementation as cost of obstetric ultrasound becomes more affordable in low- and middle-income countries. Additionally, there is a need to more clearly identify the capabilities and the limitations of ultrasound, particularly within the context of limited training of providers, to ensure that the purpose for which an ultrasound is intended is actually feasible. We found evidence of obstetric uses of ultrasound improving patient management. However, there was evidence that ultrasound use is not associated with reducing maternal, perinatal or neonatal mortality. Patients in various studies reported to have both positive and negative perceptions and experiences related to ultrasound and lastly, illegal use of ultrasound for determining fetal sex was raised as a concern.

## Background

There are numerous applications and indications for ultrasound in low- and middle-income countries (LMICs). Ultrasound has been used to diagnose obstructed labor, non-cephalic presentation, single or multiple pregnancy, incomplete miscarriage, molar pregnancy, ectopic pregnancy, fetal abnormality, intrauterine growth restriction and placenta previa [1–5]. It has also been used to measure pelvic outlet and estimate gestational age [5–7]. Although the degree of diagnostic accuracy may vary depending on when pregnant women present themselves for an ultrasound exam [7], its utility has been highlighted in many studies nonetheless. Accurate assessment of gestational age for example is useful in distinguishing pre-term newborns from newborns who are low birth weight (but not pre-term), which is important because the needed interventions may differ [8].

In LMICs, ultrasound is mainly used to diagnose obstetric conditions [7, 9]. However, gynecological conditions have also been evaluated by ultrasound as well, particularly in emergency medicine [9, 10]. Kotlyar and Moore stated that 53% of ultrasound scans were performed either for obstetric or gynecological conditions in a single center study from Monrovia, Liberia [11]. In another study in Cameroon, ultrasound determined that 33% of patients had gynecological conditions and 15% had obstetric conditions [12]. Applications of ultrasound also extend to abdominal, musculoskeletal, cardiac, renal, pulmonary, trauma and soft tissue and vascular conditions [3, 7, 10, 13, 14] as well as HIV, tuberculosis, intussusception, Wilms’ tumor, Burkitt’s lymphoma, hepatitis C, Chagas’ disease, filariasis, myiasis and other protozoal, helminthic, viral and bacterial infections [7, 9]. For example, a trial in Rwanda reported that due to the high prevalence of HIV related cardiomyopathies, TB-related pericardial effusions and rheumatic disease, cardiac applications of ultrasound were frequently used [14]. In Botswana, most of the disease burden at the district level public hospitals are obstetric-related, followed by gynecological conditions and hepato-biliary conditions [4]. In Sudan where the prevalence of schistosomiasis is high, applications of ultrasound were highest for liver and spleen disorders, followed by obstetric conditions [15]. As evidenced by past studies, applications of ultrasound use are diversified based on geographic and disease context. Such high adoptability and wide ranging applications of ultrasound make it suitable for use in LMICs.

In recent years, portable ultrasound machines have become increasingly popular in LMICs due to their affordability, user-friendliness and adoptability to the harsh and restrictive conditions of resource-poor settings [3, 6, 7, 10, 16, 17]. For example, newer ultrasound models are hand-held or hand-carried [17], and the price has significantly decreased compared to that of full-size ultrasound machines [3]. The interface software on these devices have also been simplified and streamlined [6] so that they may be used more broadly by different cadres of health workers, who have different capabilities [10]. As newer generations of ultrasound are becoming more suitable for LMICs, manufacturers could be looking to expand their markets and local healthcare staff may be weighing the benefits and disadvantages of introducing these newer ultrasound models into their facilities. Multilateral and bilateral organizations as well as private donors, therefore, may want to comprehensively assess the feasibility of introducing ultrasound in these settings before appropriating funds.

Although growing, evidence on the access, utility, effectiveness, and cost-benefit of obstetric ultrasound in resource-constrained settings is still somewhat limited [5, 18]. It is also important to note that there is no evidence of its impact on reducing maternal or perinatal mortality [9, 19, 20]. Hence, questions around the purpose and the intended benefit as well as potential challenges across various domains must be carefully reviewed prior to implementation and scale-up of obstetric ultrasound technology in LMICs.

This narrative review discusses these issues for those trying to implement or scale-up ultrasound technology in LMICs. Issues addressed in this review include health personnel capacity, maintenance, cost, overuse and misuse of ultrasound, miscommunication between the providers and patients, patient diagnosis and care management, health outcomes, patient perceptions and concerns about fetal sex determination. Because obstetric conditions are the most common indication for ultrasound in LMICs [7, 9], this review mainly focuses on obstetric uses of ultrasound.

## Methods

The existing literature on the use of ultrasound in LMICs was reviewed. The initial search was focused only on published review articles because of the short timeframe (May 3rd – August 2nd, 2016) in which to prepare for a Technical Consultation Meeting on Ultrasound Use in Low- and Middle-Income Countries which was held in Washington D.C. on August 2nd, 2016. This narrative review was initially prepared for the Technical Consultation Meeting in Washington D.C. but was later expanded to include additional articles.

**Fig. 1:**
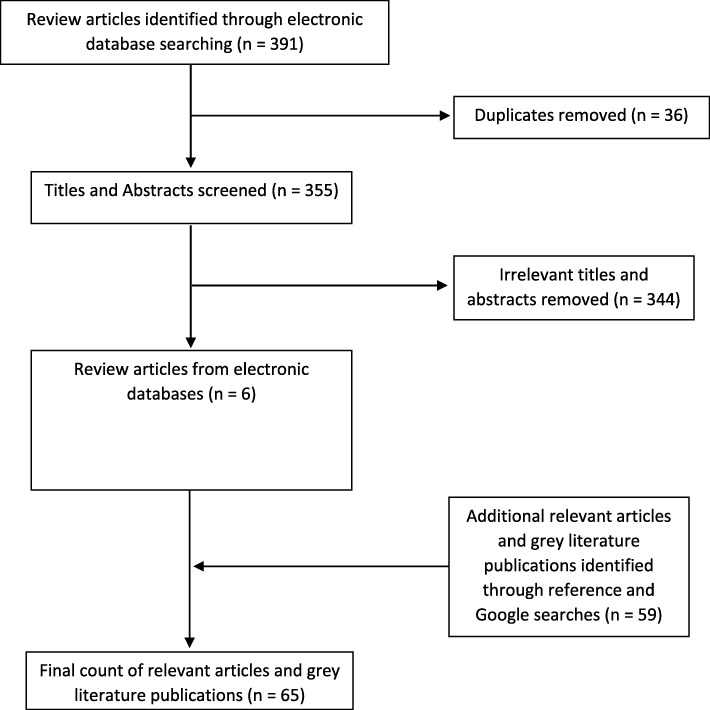
Flowchart of search results

Published review articles were searched in the following databases: PubMed, Global Health, CINAHL, Web of Science and Embase. Exact search terms and filters for the review articles are included in the Appendix. The initial database search returned a total of 391 articles and 6 review articles were retained for the literature review. After the initial electronic database search, 59 additional relevant articles and grey literature publications were identified through manual reference and Google searches. In this paper, data are synthesized from 65 sources (See Fig. 1).

Summary tables are presented for articles/studies in the review (See Tables 1, 2 and 3). Study quality was assessed using the following criteria: 1) study design –randomized or non-randomized; 2) presence of a control group. Information about the study location, study period, study description, key points, sample size, funding support and type of ultrasound machine are also presented. Qualitative studies or studies from which anecdotal accounts were retrieved were not included.

**Table 1:** Quality Assessment of Studies related to Outcome Measures

Author, Year	Location	Study Period	Type of Ultrasound	Study Description	Key Points	Sample Size	Funding or Support
Ross AB, DeStigter KK, Rielly M, Souza S, Morey GE, Nelson M, et al. 2013 [23]	Uganda	2 years	Low cost, portable ultrasound machines	Quantitative study - no control group included. Historical control data was used as a comparison. Women received ultrasound scan at the first ANC visit and at 32 weeks gestation. Based on need, ultrasound scans were performed at other ANC visits as well. Two midwives at the study health center were trained for 3 days. Training included classroom and hands-on sessions with a competency assessment. The focus of the program was on diagnosing multiple gestations, sequelae of abortion, causes of obstructed labor and placenta previa.	Total antenatal care visits significantly increased from 133.5 visits to 230.3 visits. The number of first antenatal care visits increased from 82.2 visits to 119.1 visits. The number of second antenatal care visits increased from 35.6 visits to 60.4 visits. The number of third antenatal care visits increased from 11.6 visits to 31.9 visits. The number of fourth antenatal care visits increased from 4.1 visits to 19.0 visits. The average number of deliveries at the health center significantly increased from 28.1 deliveries to 45.6 deliveries.	Prior to introduction of ultrasound: Antenatal visits (*n* = 41) Attended deliveries (*n* = 23) After introduction of ultrasound: Antenatal visits (*n* = 23) Attended deliveries (*n* = 23)	Imaging the World (received funding from the Fineberg Foundation, the Bill and Melinda Gates Foundation, Phillips Health Care, McKesson Corporation, Peervue Corporation). Research project was also supported by an Alpha Omega Alpha postgraduate award.
Mbuyita S, Tillya R, Godfrey R, Kinyonge I, Shaban J, Mbaruku G, 2015 [25]	Tanzania	1 year	Portable hand-held Vscan	Quantitative and Qualitative study - Before and After design with intervention and control groups. Portable handheld ultrasound technology (Vscan) was introduced in routine ANC services. Assistant medical officers at the health center level and mid-level providers (clinical officers, nurse midwives and medical attendants) at the dispensary level were trained to use the Vscan ultrasound in routine ANC services.	Between baseline and endline periods in the intervention area: The number of first antenatal care visits did not change significantly. The number of four or more antenatal care visits increased significantly. The number of facility deliveries increased significantly.	Intervention arm: Baseline (*n* = 381) Endline (*n* = 423) Control arm: Baseline (*n* = 394) Endline (*n* = 383)	GE Healthcare East Africa Services Limited supported the project
Van Dyk B, Motto JA and Buchmann EJ, 2007 [19]	South Africa	Not Mentioned	Not Mentioned	Quantitative study - cluster-randomized controlled trial. Control group of no ultrasound was included. Routine second-trimester ultrasound scans were performed by an experienced licensed ultrasonographer to women with low risk pregnancies at 18–23 weeks by clinical estimation.	Routine second-trimester ultrasound scanning did not result in a significant difference in perinatal mortality between the ultrasound scan group and the control group. However, sample size was insufficient.	Intervention group (*n* = 416) Control group (*n* = 388)	Not Mentioned
Geerts LT, Brand EJ and Theron GB, 1996 [22]	South Africa	10 months	Aloka SSD-500, SSD-620, SSD-650, with 3.5 MHz curvilinear electronic probes.	Quantitative study - randomized controlled trial. Control group of selective ultrasound compared to the intervention group of routine ultrasound. “Pregnant patients without risk factors for congenital anomalies referred for ultrasonography between 18 and 24 weeks of gestation” participated in the study. Women in the intervention group received “a single level one ultrasound exam performed by obstetric registrars or medical officers specifically trained in obstetric ultrasound.”	The difference in perinatal mortality between the intervention and control groups was not statistically significant. There was not enough sample size to detect differences in perinatal mortality alone. However, when combined with major morbidities, there was a significant increase (25%) in total adverse perinatal outcomes in the control group without routine ultrasonography.	Intervention group (*n* = 496) Control group (*n* = 492)	Not Mentioned
McClure E, Goldenberg R, Swanson D, Saleem S, Esamai F, Garces A, et al. 2017 [20]	Pakistan, Kenya, Zambia, Democratic Republic of Congo, Guatemala	18 months	Ultrasound equipment donated by GE Healthcare	Quantitative study - cluster randomized trial. Clusters were randomized either to the control group of usual care or to the intervention group of receiving ultrasound at 16–22 and 32–36 weeks and appropriate referrals. Multiple intervention components were included: Two-week obstetric ultrasound training for varying cadres of non-physician health workers. 3-month pilot period and follow-up quality assurance system to ensure successful implementation. Training for referral facility staff in management of major obstetric and neonatal conditions. Meetings with ministry of health officials and hospital administrators in order to improve health system management. Community orientations in order to raise awareness about the availability of service and the purpose of ultrasound.	There was no difference in primary composite outcome, stillbirth rate, neonatal mortality rate, near miss rate, maternal death, at least one antenatal visit, four or more antenatal visits and delivery at the hospital with cesarean section capacity between the intervention and control groups. 78% of deliveries in the intervention group received one or more ultrasound service and 60% received two ultrasound services. 9% of women were referred based on ultrasound diagnosis and 71% followed up with the referrals.	Guatemala (18 clusters) Zambia (10 clusters) Kenya (12 clusters) Pakistan (10 clusters) Democratic Republic of the Congo (8 clusters)	Trial funded by Bill & Melinda Gates Foundation. The ultrasound trial conducted by Global Network for Women’s and Children’s Health Research (supported by Eunice Kennedy Shriver National Institute of Child Health and Human Development). Obstetric ultrasound training oversight by The University of Washington Department of Radiology with support from GE Healthcare
Ross AB, DeStigter KK, Coutinho A, Souza S, Mwatha A, Matovu A, et al. 2014 [26]	Uganda	2 years	Low cost, portable ultrasound machines	Quantitative study - no control group included. Historical control data was used as a comparison. Women received ultrasound scan at the first ANC visit and at 32 weeks gestation. Based on need, ultrasound scans were performed at other ANC visits as well. Two midwives at the study health center were trained for 3 days. Training included classroom and hands-on sessions with a competency assessment. The focus of the program was on diagnosing multiple gestations, sequelae of abortion, causes of obstructed labor and placenta previa.	Comparing post-intervention ultrasound group to pre-intervention historical control: The relative rate ratios for anemia treatment, RR = 1.26 (1.15–1.38); deworming treatment, RR = 1.21 (1.11–1.32); and IPT2 treatment, RR = 2.13 (1.35–3.35) were all significantly higher than 1. The relative rate ratio for HIV testing was not statistically significant and the relative rate ratio for IPT1 treatment, RR = 0.88 (0.79–0.98) was significantly lower than 1.	Prior to introduction of ultrasound: Anemia – 34 months Deworming – 34 months HIV testing – 24 months IPT1–40 months IPT2–36 months After introduction of ultrasound: Anemia – 22 months Deworming – 23 months HIV testing – 23 months IPT1–23 months IPT2–23 months	Study supported by Bill and Melinda Gates Foundation, Sanofi, Philips Healthcare, McKesson Corporation and the Fineberg Foundation

**Table 2:** Summary of Studies on Ultrasound’s Impact on Patient Care Management

Author, Year	Location	Study Period	Type of Ultrasound	Study Description	Key Points	Sample Size	Funding or Support
Bussmann H, Koen E, Arhin-Tenkorang D, Munyadzwe G and Troeger J, 2001 [4]	Botswana	18 months	Real-time ultrasound scanner Siemens SL-1, with a 3.5 MHz/5 MHz sector probe and a 5 – MHz linear probe.	Quantitative study - no control group included. Clinical diagnosis compared with ultrasound-aided diagnosis without blinding. Clinicians made a provisional clinical diagnosis. Then, the sonographer made an ultrasound-aided diagnosis with knowledge of the clinical diagnosis. Patients were enrolled based on a pre-developed indication list. The list included conditions relevant to the study hospital where ultrasound diagnosis could be useful: obstetric, cystic and solid masses differentiation, urinary or biliary obstruction, detection of fluid in peritoneal, pleural or pericardial cavity or ventricular brain space, etc. Diagnosis by ultrasound was categorized as either “similar improved”, “different improved” or “not improved”. “Similar improved” indicated ultrasound providing a diagnosis of high certainty when clinical diagnosis was of low certainty. “Different improved” indicated ultrasound providing a diagnosis of high certainty when clinical diagnosis had not been ascertained. “Not improved” indicated ultrasound diagnosis being of equal certainty with clinical diagnosis or if no pathology was found.	Overall, 334 of 722 (45%) of all pregnant cases had improved diagnosis due to ultrasound examination. Of the 334 improved cases, 201 (27%) were categorized as “similar improved” and 133 (18%) were categorized as “different improved”. For 49 cases, care management changed immediately after the ultrasound diagnosis. This study showed that ultrasound examinations can aid clinical diagnosis and thereby contribute to improving the quality of care in a district hospital.	2309 patients	Life Sciences and Technology for Developing Countries program of the Commission of the European Communities supported the study.
Kimberly HH, Murray A, Mennicke M, Liteplo A, Lew J, Bohan JS, et al. 2010 [6]	Zambia	6 months	SonoSite 180 portable ultrasound, with an extra battery, a curved array abdominal probe and software for image storing	Quantitative study - no control group included. Observed examinations conducted at midterm and at the end. “Most recorded scans were performed in second and third trimester.” 21 midwives underwent three training periods which were 2 to 3 weeks in length and were separated by 2 to 3 months of independent scanning. Training took place in the form of didactic sessions, practical hands on sessions and supervised scanning. The training content included identification of fetal presentation, fetal heart rate, placental location, multiple gestations, and estimation of gestational age based on biparietal diameter and femur length.	From a total of 441 ultrasound scans that were performed, 74 ultrasound scans (17%) led to a change in clinical decision-making. Of the cases that had a change in clinical management, non-vertex presentation, multiple gestations, lack of fetal heart rate and low lying placenta consisted over 95% of the findings.	441 scans performed	SonoSite donated ultrasound machines
Kotlyar S and Moore CL, 2008 [11]	Liberia	5 weeks	A portable LOGIQe US unit, with a 2.5-MHz curvilinear probe, a 5-MHz phased array cardiac probe, a 7-MHz linear probe, and a 4.5-MHz endovaginal probe. Doppler and color flow capabilities were available.	Quantitative study - no control group included. Pre-ultrasound diagnosis compared with After-ultrasound diagnosis. “Ultrasound images were acquired by residency trained emergency physicians as part of routine patient care. Upon physicians’ requests, ultrasound was performed on inpatients in the medical, pediatric, surgical and obstetrical wards as well as patients in the emergency room.”	Ultrasound examination changed patient management in 62% of cases. Of the cases that had a change in patient management, the highest impact was seen with first trimester obstetric conditions, focused assessment of sonography in trauma exam, cardiac applications and second and third trimester obstetric conditions.	102 patients	GE Medical loaned ultrasound equipment
Stein W, Katunda I and Butoto C, 2008 [27]	Tanzania	12 months	Siemens SL-1, with a 3.5 MHz/5 MHz sector probe, 8 MHz vaginal probe.	Quantitative study - no control group included. Clinical diagnosis compared with ultrasound-aided diagnosis. The first-level ultrasound was randomly conducted on patients with absent fetal heartbeat and suspect fetal position other than cephalic. For suspected twins and vaginal bleeding, it was regularly re-examined. The second-level advanced ultrasound was performed by a specialist sonographer for patients with fundal height discrepancies, suspected incomplete abortion, suspected extra uterine pregnancy and abdominal pain. Midwives received two months of training in basic obstetric ultrasound to perform first-level ultrasound services.	During the study period, 542 patients were enrolled. Of which, ultrasound diagnosis improved clinical management in 212 (39.1%) of the cases, conflicted with original clinical diagnosis in 81 cases (14.9%) and led to a change of management in 121 cases (22.3%).	542 patients	Not Mentioned
Shah SP, Epino H, Bukhman G, Umulisa I, Dushimiyimana JM, Reichman A, et al. 2009 [14]	Rwanda	19 weeks +	Sonosite Micromaxx, with endocavitary probe, a curved array abdominal probe and cardiac probes	Quantitative study - no control group included. Blinded review of ultrasound scans for accuracy and quality. Datasheets for each ultrasound scan performed during routine clinical care were collected and analyzed. Local physician staff underwent a 9-week ultrasound training curriculum that included lectures and practical hands-on sessions. Lecture topics included obstetrical ultrasound, cardiac ultrasound, hepato-biliary ultrasound and other advanced uses of ultrasound.	Providers at the study sites indicated that ultrasound scans changed their initial patient management plan in 43% of the cases. The most commonly reported change after the ultrasound was the consideration to perform a surgical procedure such as a cesarean section, biopsy or minor surgery. Cesarean sections were decided if the patient had breech presentation, placenta previa or multiple gestations.	345 ultrasound scans	SonoSite donated ultrasound machines
Wylie BJ, Kalilani-Phiri L, Madanitsa M, Membe G, Nyirenda O, Mawindo P, et al. 2013 [8]	Malawi	4 months +	SonoSite 180 portable ultrasound	Quantitative study - no control group included. Comparison of ultrasound-aided dating and menstrual gestational age/postnatal Ballard estimation. “61.8% of the subjects were enrolled and imaged between 20 and 27 weeks. 21.3% were imaged after 28 weeks. 16.3% were imaged prior to 20 weeks.” 8 trainees (four research staff, one nurse, three midlevel clinicians) received an intensive one-week ultrasound training for fetal biometry. Four months of additional practice and remote image review followed.	Ultrasound scans were used to confirm menstrual gestational age in 62.1% of the cases. 13% were not aware of their last menstrual dates and thus had their gestational age calculated solely by ultrasound. 24.9% of the cases had to have their gestational age re-dated by ultrasound. For over a third of the patients, ultrasound scans played a critical role in improving the accuracy of their gestational age. Because Malaria during pregnancy is a risk factor for low birth weight, accurate dating by ultrasound can facilitate appropriate clinical management	178 patients	Ultrasound machines donated by Vincent Department of Obstetrics and Gynecology at the Massachusetts General Hospital with a matching grant from the SoundCaring Program (Sonosite). Research supported by the Doris Duke Charitable Foundation and by the National Institute of Health.
Spencer JK and Adler RS, 2008 [28]	Ghana	1 month	A LOGIQ Book portable ultrasound, with a linear L5–10 MHz and curvilinear C3–5 MHz broadband transducers.	Quantitative study - no control group included. Determination of whether ultrasound aided clinical diagnosis. Patients were seen by a radiologist trained in general abdominal ultrasound, obstetric ultrasound, small parts and musculoskeletal ultrasound, and other procedures by ultrasound in a variety of clinical settings: a surgeon’s office, hospital operating room and clinics. Diagnoses included both infectious and non-infectious diseases.	Of the total 67 ultrasound scans performed, 54 (81%) of them were considered to be abnormal findings. All of the abnormal findings enhanced clinical diagnosis and 40% either influenced the outcome or the clinical decision-making regarding treatment.	67 patients	GE Healthcare provided a LOGIQ Book portable ultrasound
Steinmetz JP and Berger JP, 1999 [12]	Cameroon	16 months	Multipurpose ALOKA 256, with two linear probes of 3.5 and 5 MHz	Quantitative study - no control group included. Comparison between those in which the diagnosis by ultrasonography was confirmed by a certified diagnosis and those in which the diagnosis by ultrasonography could not be confirmed. Ultrasound exams were requested by a nurse or a doctor and performed by one surgeon-echographist. Indications for ultrasound exams included gynecology, liver, spleen and biliary tract, gastrointestinal tract, pregnancy, renal-urinary tract and others.	In the retrospective review of 1119 ultrasound scans, 78% of the scans were deemed as abnormal findings. 67.8% were judged useful in clinical diagnosis and management and only 4.6% were judged to be erroneous. In addition, for nearly a half of the cases that had been confirmed by tissue pathology, additional imaging tests, endoscopy, surgical specimen or laboratory diagnosis, ultrasound scans suggested a differential diagnosis which had not been previously considered.	1119 ultrasound scans	Samaritan Hospital provided the ultrasound machine. Foundation de Jumelage Oona Chaplin provided funding support.
Shorter M and Macias DJ, 2012 [16]	Haiti	12 days	Signos handheld ultrasound, with a 3.5 MHz and a 7.0 MHz removable probe.	Quantitative study - no control group included. Retrospective, non-blinded, observational analysis of ultrasound scans. Ultrasound was performed by one registered diagnostic medical sonographer-eligible emergency physician sonographer (with only 8 h of prior experience with the handheld device) “Ultrasound was performed for complaints or symptoms indicative of illnesses that could potentially be triaged or diagnosed by ultrasound (internal bleeding, pregnancy, shock and cardiac dysfunction).”	The use of portable hand-held ultrasound devices led to a change in 70% of patient management plans. Portable ultrasound imaging was found especially useful for non-traumatic abdominal pain and pregnancy-related symptoms.	50 patients	Not Mentioned

**Table 3:** Summary of the Literature on Ultrasound Training Programs

Author, Year	Location	Study Period	Type of Ultrasound	Study Description	Key Points	Sample Size	Funding or Support
Stanton K and Mwanri L, 2013 [7]	Low resource settings	Not Applicable	Not Mentioned (Varies by Study)	Systematic review.	Midwives and birth attendants can be trained in ultrasound	32 articles	Not Mentioned
LaGrone LN, Sadasivam V, Kushner AL and Groen RS, 2012 [18]	Low- and middle- income countries	Not Applicable	Not Mentioned (Varies by Study)	Review article.	Generalist and obstetric physicians perform most ultrasound scans. The types of ultrasonography training ranged from “no formal training to formal certification and residency programs.” (p. 808) All programs had courses that consisted of didactics and hands-on sessions. The types of follow-up training ranged from “none, to telemedicine case review, to formal re-evaluations and intensive refresher courses.” (p. 809) Although ultrasound training in LMICs often does not meet the WHO criteria, select programs reported high levels of diagnostic accuracy and trainees’ knowledge retention.	41 articles for final review	Not Mentioned
Carrera JM, 2011 [31]	Africa	Not Applicable	Not Mentioned (Varies by Study)	Review article.	Most practitioners in Africa are not well-trained in ultrasonography. 40.4% have only received a theoretical short course. 38.3% did not have training. 14.9% received a practical course.	Not Mentioned	Not Mentioned
Kimberly HH, Murray A, Mennicke M, Liteplo A, Lew J, Bohan JS, et al. 2010 [6]	Zambia	6 months	SonoSite 180 portable ultrasound,with an extra battery, a curved array abdominal probe and software for image storing	Quantitative study - no control group included. Observed examinations conducted at midterm and at the end. “Most recorded scans were performed in second and third trimester.” (p. 1269) 21 midwives underwent three training periods which were 2 to 3 weeks in length and were separated by 2 to 3 months of independent scanning. Training took place in the form of didactic sessions, practical hands on sessions and supervised scanning. The training content included identification of fetal presentation, fetal heart rate, placental location, multiple gestations, and estimation of gestational age based on biparietal diameter and femur length.	96% agreement between expert reviewers and trained midwives on Interpretation of fetal heart rate. 91% agreement between expert reviewers and trained midwives on identification of placental location. 70% disagreement between expert reviewers and trained midwives on measurement of biparietal diameter. Clinical decision-making changed in 17% of the cases.	441 scans performed	SonoSite donated ultrasound machines
Wylie BJ, Kalilani-Phiri L, Madanitsa M, Membe G, Nyirenda O, Mawindo P, et al. 2013 [8]	Malawi	4 months +	SonoSite 180 portable ultrasound	Quantitative study - no control group included. Comparison of ultrasound-aided dating and menstrual gestational age/postnatal Ballard estimation. “61.8% of the subjects were enrolled and imaged between 20 and 27 weeks. 21.3% were imaged after 28 weeks. 16.3% were imaged prior to 20 weeks.” 8 trainees (four research staff, one nurse, three midlevel clinicians) went through an intensive one-week ultrasound training for performing fetal biometry. Four months of additional practice and remote image review followed.	Only 5.7% of the ultrasound scans with a biometric parameter were considered unacceptable. Ultrasound helped improve gestational age dating in over a third of the research subjects (compared to menstrual dating). The median gestational age determined by ultrasound dating and by postnatal Ballard estimation were significantly different. The trainees did not become proficient immediately after the first one-week training course. An additional four months of practice and remote image review were paramount in achieving the necessary skill.	178 patients	Ultrasound machines donated by Vincent Department of Obstetrics and Gynecology at the Massachusetts General Hospital with a matching grant from the SoundCaring Program (Sonosite). Research supported by the Doris Duke Charitable Foundation and by the National Institute of Health.
Rijken MJ, Lee SJ, Boel ME, Papageorghiou AT, Visser GH, Dwell SL, et al. 2009 [32]	Thai-Burmese border	6 months	Toshiba Powervision 7000 machine with a 3.75-MHz convex probe	Quantitative study - no control group included. Intraobserver and interobserver variations measured. Ultrasound was performed on every fifth pregnant woman with a singleton pregnancy between 16 and 40 weeks’ gestation and who had undergone an early dating ultrasound scan, attending the antenatal clinic. Four local health workers received a 3-month course consisting of practical and theoretical training in obstetric ultrasound. The training curriculum was developed based on the World Health Organization guidelines and British Medical Ultrasound Society recommendations.	Compared with the skilled sonographer, the trainees’ fetal anthropometric measurements showed high level of agreement.	349 patients	Ultrasound scanner donated by Ph. Stoutenbeck and the Department of Obstetrics of the University Medical Center Utrecht, The Netherlands. NIHR Biomedical Research Program funded A.T.P.
Kozuki N, 2015[5] Kozuki N, Mullany LC, Khatry SK, Ghimire RK, Paudel S, Blakemore K, et al. 2016 [52]	Nepal	7 months	Sonosite Nanomaxx system, and a C60n (obstetric) probe	Quantitative study - control group included for comparing facility delivery rates and rate of adverse outcomes. Pregnant women who were gestational age 32 weeks or more and consented to participate in the study were visited in their homes. Three auxiliary nurse midwives received two one-week ultrasound training sessions which were separated by a month. Training took place in the form of lectures, demonstrations and practice. The training content included identification of fetal presentation, fetal heartbeat, multiple gestation and placental position.	The “cannot determine” selection by two reviewer teams for fetal presentation was 0.1 and 0.3% of the ultrasound exams. The “cannot determine” selection by two reviewer teams for multiple gestation was 0.9 and 6.6% of the ultrasound exams. The “cannot determine” selection by two reviewer teams for placenta previa was 34 and 44% of the ultrasound exams.	804 women	Children’s Prize, the National Institutes of Health/ National Institute of Child Health and Human Development, Bill and Melinda Gates Foundation. Ultrasound machines donated by SonoSite Soundcaring Program.
Stein W, Katunda I and Butoto C, 2008 [27]	Tanzania	12 months	Siemens SL-1, with a 3.5 MHz/5 MHz sector probe and a 8 MHz vaginal probe	Quantitative study - no control group included. Clinical diagnosis compared with ultrasound-aided diagnosis. The first-level ultrasound was randomly conducted on patients with absent fetal heartbeat and suspect fetal position other than cephalic. For suspected twins and vaginal bleeding, it was regularly re-examined. The second-level advanced ultrasound was performed by a specialist sonographer for patients with fundal height discrepancies, suspected incomplete abortion, suspected extra uterine pregnancy and abdominal pain. Midwives received two months of training in basic obstetric ultrasound to perform first-level ultrasound services.	Trained midwives identified twins, fetal heart rate and fetal position with 100% agreement as the sonographer. The results for vaginal bleeding agreed in 76.6% of the cases between midwives and the sonographer.	542 patients	Not Mentioned
Bell G, Wachira B and Denning G, 2016[33]	Kenya	15 months	Not Mentioned	Quantitative study - no control group included. Trainees’ mean scores were compared across sessions. Trainees were given materials to study before the workshop. They then participated in three all-day workshops held every 3 to 5 months. Training included review of content for point-of-care ultrasound (“the abdominal, pleural and cardiac assessment for free fluid, the thoracic exam for pneumothorax, an obstetric exam for intrauterine pregnancy, cul-de-sac fluid, fetal heart activity and position” - p. 2), demonstrations and practice. Professional cadres of trainees included clinical officers, doctors and nurses.	After the workshop, participants demonstrated increase in knowledge and acquisition of practical skills. Average scores for practical skills were not significantly different among varying cadres of healthcare workers.	81 trainees	The Christian Health Association of Kenya provided training facilities, DAK Foundation provided ultrasound equipment, The Emergency Medicine Kenya Foundation, The Emergency Medicine Department at the University of Iowa and The Aga Kahn University Hospital provided funding support.
Greenwold N, Wallace S, Prost A and Jauniaux E, 2014 [34]	Mozambique	12 months	Sonosite 2 M-Turbo portable ultrasound, with integrated obstetric biometric charts	Quantitative study - no control group included. Detection rates for trainees under supervision and without supervision were compared. All women attending prenatal care between 11 weeks and term were offered ultrasound exams. 9 nurses and clinical officers underwent an 8-week training course followed by 10 months of remote supervision. Training included 1 week of formal lectures and 7 weeks of practical hands-on sessions. The training content included basic uses of ultrasound, first-trimester ultrasound, estimation of gestational age and identification of fetal presentation, placental position, multiple pregnancies and uterine fibroids.	Comparison of the detection rates for basic ultrasound findings between the two groups reveals that fetal anomalies are the only condition that the participants under direct supervision detected significantly higher. The detection rates for pregnancy loss/intrauterine fetal death, twin pregnancies, fibroids, placenta previa, breech presentation and transverse presentation were statistically similar between the two groups.	1744 pregnant women, 804 scanned images by trainees under direct supervision, 940 scanned images by trainees alone	Medical Aid Films, MaMA Mozambique and Sonosite provided financial support
Shah SP, Epino H, Bukhman G, Umulisa I, Dushimiyimana JM, Reichman A, et al. 2009 [14]	Rwanda	19 weeks +	Sonosite Micromaxx, with endocavitary probe, a curved array abdominal probe and cardiac probes	Quantitative study - no control group included. Blinded review of ultrasound scans for accuracy and quality. Datasheets for each ultrasound scan performed during routine clinical care were collected and analyzed. Local physician staff underwent a 9-week ultrasound training curriculum that included lectures and practical hands-on sessions. Lecture topics included obstetrical ultrasound, cardiac ultrasound, hepato-biliary ultrasound and other advanced uses of ultrasound.	Between the participants and the ultrasound-trained emergency medicine physicians, there was 96% agreement in their interpretations of the scans. Participants continued to use ultrasound after the training.	345 ultrasound scans	SonoSite donated ultrasound machines
Sippel S, Muruganandan K, Levine A and Shah S, 2011 [10]	Developing world	Not Applicable	Not Mentioned (Varies by Study)	Review article.	“No standardized approaches available for the length of training, curriculum for general practitioners, qualifications of trainers or mechanism of training.” (p. 4) The length of ultrasound training ranged between 4 days and several months. The cadres of trainees ranged from clinical officers, nurses and nurse midwives to physicians. Trainers ranged from resident physicians, emergency physicians, radiologists, cardiologists to ultrasound-fellowship trained emergency physicians. The curriculum and method of training varied based on the specific goals of the programs. Overall, “a short but intensive training period is sufficient for preparing clinical officers, nurses and physicians alike to perform basic ultrasound exams” (p. 4), especially when both didactics and practical sessions are included and skills maintenance is ensured through refreshers.	Not Mentioned	Not Mentioned
Becker DM, Tafoya CA, Becker SL, Kruger GH, Tafoya MJ and Becker TK, 2016 [17]	Low- and middle-income countries	Not Applicable	Varies by Study	Systematic review.	“Short-training courses may lead to significant knowledge retention and improve practical skills, even when prior ultrasound experience was minimal.” (p. 307)	36 manuscripts included for final review	Not Mentioned
Swanson JO, Kawooya MG, Swanson DL, Hippe DS, Dungu-Matovu P and Nathan R, 2014 [35]	Uganda	10 months	GE Logiq E Ultrasound and GE Logic Book XP, with wide-band (2.0 to 5.5 MHz) convex array transducers	Quantitative – no control group included. Expecting mothers received ultrasound exams as part of the routine antenatal care visit. 14 midwives from the study health centers underwent a 6-week ultrasound training course on limited obstetric ultrasound. 13 of which had no prior ultrasound training. The training curriculum included “ultrasound physics, relevant anatomy and physiology, instrumentation and basic maintenance.” (p. 509) Training methods included “lectures, small-group tutorials, audiovisual materials and supervised clinical scanning.” (p. 509)	Obstetric ultrasound provided by midwives changed the clinical diagnosis in up to 12% of the cases. The quality assurance review of midwives’ scans diagnosing gestational number showed 100% sensitivity and specificity. The quality assurance review of midwives’ scans diagnosing fetal presentation showed 90% sensitivity and 96% specificity.	939 patients	GE Foundation, University of Washington Radiology Health Services Research Seed Grant Program and Seattle International Foundation supported the study.

## Findings

### What are the effects of obstetric ultrasound?

#### Care utilization and mortality

A recent cluster-randomized trial in Pakistan, Kenya, Zambia, the Democratic Republic of the Congo and Guatemala looked at the effects of antenatal ultrasound use in rural health center settings [20, 21]. After stratifying by country, each of the intervention and comparison clusters were defined as a catchment area of a health center that records about 500 births every year [21]. Pregnant women in the intervention clusters were generally offered two antenatal ultrasound examinations, first exam between 18 and 22 weeks and the second exam between 32 and 36 weeks, and those with identified complications were referred to higher-level health facilities [21]. At the first visit, gestational age was determined and fetal number and position, cervical length, potential amniotic fluid abnormalities and potential congenital abnormalities were examined [21]. At the second visit, placental location and growth were observed in addition to all of the checks performed during the first visit [21]. The intervention package also included health worker training for antenatal ultrasound, emergency obstetric and neonatal care training at higher-level referral facilities and community sensitization events where people were informed about the diagnostic capabilities of ultrasound as well as the antenatal ultrasound services being offered at intervention facilities [21]. This recent study including five countries with a rigorous cluster-randomized design found no difference in intervention and comparison clusters in terms of antenatal care use, facility delivery, stillbirth rate, neonatal mortality and maternal mortality [20].

Another randomized study conducted in South Africa found that routine second-trimester ultrasound scanning did not result in a significant difference in perinatal mortality between the ultrasound scan group and the control group, 4.3 and 4.1% respectively with a relative risk of 1.05 and 95% confidence interval between 0.54 and 2.03 [9, 19]. However, the study also noted that it had low power due to the small sample size [19]. Other studies either did not have enough statistical power to detect significant differences in mortality outcomes [22] or simply speculated that increases in antenatal care visits as a result of ultrasound or postpartum uses of ultrasound will likely reduce mortality outcomes [23, 24].

Some studies that employed a weak study design (limited randomization and absence of control groups) reported that ultrasound services generally appeared to be associated with an increase in the number/proportion of antenatal care use and facility delivery, which is contrary to the findings of a recent large-scale cluster-randomized trial [20, 21].

Ross et al. conducted a study at a rural community health center in Uganda with portable ultrasounds [23]. Exams were offered to women during their first antenatal visit and at 32 weeks gestation, and additional scans were performed when they were deemed appropriate [23]. The study noted that ultrasound exam fees were affordable to all patients at about $2 USD [23]. Lacking a control group, the study utilized a historical control to assess changes in outcome measures pre and post-intervention [23]. Total antenatal care visits at the health center significantly increased from a monthly average of 133.5 visits before the program to 230.3 visits after the program [23]. Over a period of 64 months, the average number of deliveries at the health center also significantly increased from 28.1 deliveries before the program to 45.6 deliveries after the program [23].

Mbuyita et al. conducted a study in Tanzania to determine if mid-level providers were capable of using portable ultrasound machines after training [25]. Compared to baseline data, the number of first antenatal care visits did not change significantly [25]. However, the number of women receiving four or more antenatal care visits increased significantly between baseline (27.2%) and endline (60.3%) periods in the intervention area [25]. The number of facility deliveries also increased significantly between baseline and endline periods in the intervention area [25].

Lastly, Ross et al. examined the components of routine antenatal care services before and after the introduction of routine ultrasound [26]. The study showed significantly increased rates of anemia treatment, deworming, and two doses of intermittent preventive treatment of malaria (IPT2) after the ultrasound intervention [26].

#### Patient diagnosis and care management

Although recent evidence points to ultrasound having no significant effect on mortality, facility delivery and antenatal care use [20, 21], there were studies reporting that ultrasound in LMICs helped improve the quality of care for both obstetric and non-obstetric conditions [10]. Muller-Rockstroh, in her narrative description about a hospital in Northwest Tanzania, stated that ultrasound scans helped midwives determine the timing and appropriate mode of delivery. Midwives were able to delay or accelerate timing of delivery and determine appropriate management plans based on ultrasound findings [24, 25]. The ability of ultrasound to confirm clinically suspected obstetric complications and even improve patient management has been reported in many other studies [4, 6, 8, 11, 12, 14, 27, 28].

A study in Malawi reported that despite having a learning curve, mid-level providers were able to confirm or improve determination of gestational age using ultrasound [8]. Correct determination of gestational age is necessary to distinguish premature newborns from newborns who are growth-restricted as the required care and interventions are different [8]. Correct determination of gestational age also has implications for preventing mother-to-child transmission of HIV [7].

The utility of ultrasound has been documented in medical emergencies and disaster settings which include obstetric uses as well. Understandably, portable hand-held or hand-carried ultrasound devices have been employed in these situations where there is unreliable access to power and a high need for rapid triage [17]. During a 12-day disaster relief effort in Haiti after the earthquake in 2010, the use of portable hand-held ultrasound devices led to a change in 70% of patient management plans [16]. Portable ultrasound imaging was found especially useful for diagnosing non-traumatic abdominal pain and pregnancy-related symptoms [16].

### What are key issues related to health personnel?

#### Training of health personnel

The training of health personnel is a major factor for quality implementation of obstetric ultrasound services, as appropriate patient management relies on the ability of health personnel to use the machine proficiently and interpret findings accurately [7]. In many LMICs, there is a paucity of health personnel who are capable of providing ultrasound services [29].

A WHO Study Group recommended in its 1998 ultrasound manual that candidates for ultrasound training should have at least two to three years of prior healthcare training [30]. Qualified candidates are then recommended to complete at least 6 months of training in a recognized training center including 50 first trimester pregnancy exams and 200 s and third trimester exams for obstetric applications of ultrasound [30]. It is important to note, however, that recommendations for the different numbers of examinations were given as a guide. A recent review article, which documented training opportunities for ultrasonography in LMICs, concluded that the majority of health personnel using ultrasound in LMICs did not meet the minimum WHO training standards [18]. In Africa, 40.4% of ultrasound service providers completed only a short theoretical course, 14.9% had practical courses with only 2.1% occurring in a hospital environment, and 38.3% had no training at all [7, 31]. Ultrasonography training largely targeted lower- and mid-level practitioners such as local health workers, nurses and midwives as well as non-radiologist physicians [18]. Although the literature suggests that past trainings were not in compliance with WHO’s recommendations, many studies demonstrated that lower- and mid-level providers were capable of providing ultrasound services resulting in improved clinical management [5, 6, 8, 14, 27, 29, 32–35]. There is also some evidence that general physicians at rural health facilities can adequately provide ultrasound services in terms of correct use and interpretation of findings [14].

When adequate training materials and methods are used, short intensive training courses have been shown to provide significant acquisition of knowledge and practical skills for all levels of health workers in LMICs [10, 17]. There is also evidence that follow-up refresher trainings can be effective for retention of knowledge and skills [10, 17] though the sustainability of these courses after donor funding is expended is not adequately documented. In addition to training, however, it is important to note that there are other issues which are essential in obtaining good clinical readings. These issues include ensuring that the machine is set for the proper application (i.e. obstetric uses) and that the use setting has appropriate lighting, temperature, electrical supply and IT requirements [36]. It is also important to understand the limitations of different ultrasound models and devices. For example, the image quality of portable ultrasound may not be sufficient to identify fetal anomalies and early gestational age.

#### Concerns about misuse, overuse and miscommunication

Misuse, overuse and lack of communication between health providers and patients have been frequently cited as concerns in the literature. A study in Uganda demonstrated that over half of all obstetric scans performed were considered inappropriate because they were either scans for gestational age estimation outside of the recommended time period, routine monitoring despite no sign of intrauterine growth restriction or re-assessment of the placental position because of the technical inability to determine it with a previous scan [37]. In another study in Botswana, health providers noted that conventional methods such as history taking and physical examination were neglected because of easy access to ultrasound services [2]. There were indications that even in situations where conventional methods would have sufficed, ultrasound diagnosis was recommended for convenience [2]. Such inappropriate use raises concerns because health providers should not neglect conventional methods of providing care [2, 3].

Even when ultrasound services are available, they must be complementary to routine care [4, 7]. This is especially true for obstetric use of ultrasound. For example, estimation of gestational age is more accurate early in pregnancy [8]. As pregnancy progresses, the variation of fetal size increases and it becomes difficult to accurately estimate gestational age by ultrasound alone [8]. Since pregnant women in LMICs often present themselves for antenatal care later in pregnancy, ultrasound dating must be used in conjunction with conventional methods to accurately estimate gestational age [8].

As for cases of misuse, financial incentives for providing unnecessary ultrasound services have been reported as one reason for misuse in LMICs. After the initial ultrasound scan, multiple follow-up scans were often scheduled in private clinics as a way to increase revenue [38, 39]. Using ultrasound for financial gain or any other reason that is not clinically-based, can significantly reduce the cost-effectiveness of services [1] and drive up the costs paid by women and their families.

Lastly, there is evidence for lack of communication between health providers and patients. A study in Botswana reported that the average time of interaction between providers and patients was 15 min, of which only 15% were devoted to communicating with the patient [2]. Health providers were preoccupied with the technical aspects of performing an ultrasound scan and patients received very little attention throughout the process [2]. In a study in Iran, none of the study patients reported receiving written information about the purpose of the ultrasound exam, 48% reported that the ultrasound operator did not answer any questions and 90% reported that they were never shown the image of the fetus on the ultrasound screen [40]. These findings are of concern as patients’ perception of and experience with ultrasound are mainly determined by the quality of their interaction with the health providers [2].

#### Concerns about fetal sex determination

Prior studies in different countries document that ultrasound has been illegally used for fetal sex determination. In India, the male-dominant sex ratio for children under 5 years is thought to be associated with fetal sex determination and sex-selective abortions [1]. A population-based study in Delhi, India found that 56% of respondents either did not know that fetal sex determination was illegal or thought that it was lawful [9, 41]. In the same study, 2.6% of respondents had ever requested fetal sex determination and when the female sex of the fetus was disclosed, over 63% of them were aborted [9, 41]. In a study in Nepal, 6.8% of surveyed pregnant women received ultrasound exams for fetal sex determination despite it being unlawful [42]. The study also found that compared to women who had at least one live born son prior to the recent pregnancy, women with no live born sons and three or more live born daughters had 1.55 higher odds of receiving an ultrasound exam, which may suggest use for fetal sex determination [42]. Other popular reasons for determining fetal sex were reported to be curiosity and preparation for the baby [43, 44].

Negative consequences of fetal sex determination go beyond obvious legal and ethical violations. Incorrect assessment of the fetal sex can also result in negative experiences for women, especially in settings where son preference is high. Incorrect assessment of the fetal sex is not uncommon. In a study in Ghana, fetal sex was accurately determined in only 86.5% of the cases [45]. A study in Nigeria reported that incorrect assessment of the fetal sex, particularly for mistaking female fetuses as males, resulted in marital conflict, physical violence from husbands, regret of undergoing tubal ligation, negative perception towards ultrasound and negative feelings towards the newborns [46]. Such negative consequences can be exacerbated when accreditation and regulation for ultrasound use are limited [9, 47]. Ethical training for health providers on appropriate uses of ultrasound and regulations for both public and private clinics are important [7].

### What issues are important to patients?

#### Patient perception on ultrasound services

In the studies reviewed, women in LMICs generally held positive views about ultrasound services [23]. Rijken et al. found that on the Thai-Burmese border, ultrasound was considered a tool that could increase safety during pregnancy and childbirth [23, 48]. In a rural Botswana district hospital, pregnant women showed signs of trusting the ultrasound results more than their own bodily sensations to confirm a live fetus [2]. The women expressed relief that “there was life in the baby” (p. 697) [2]. These women also referred to the ultrasound experts as the “Whites” (p. 697) and regarded the same ultrasound services provided by African health providers as substandard [2].

Cultural resistance to ultrasound services was not reported to be a major problem. A study in Zambia reported that patients would often wait in line for ultrasound exams, even after clinic hours were over [6]. In addition, many patients who came for antenatal visits stated that availability of ultrasound was their motivation for attendance [6]. A qualitative study in Tanzania reported that the majority of women desired to receive ultrasound exams despite the lack of understanding about the benefits and the procedures [49]. Those who did not view ultrasound favorably thought that ultrasound would cause harm to the fetus [49].

Negative perceptions or misconceptions about ultrasound were reported in other studies as well [40, 50]. Women interviewed in a study in rural Kenya perceived that undergoing an ultrasound exam meant there was an emergency or a problem with the pregnancy [50]. However, all interviewed women perceived ultrasound to add significant value in reassuring the health and progress of the baby [50]. In another study in Iran, 39% of women chose not to undergo an ultrasound exam due to the following reasons: ultrasound can be harmful (especially with regards to fetal malformation); results are not important; exams are expensive; and busy schedules [40].

Motivation for seeking ultrasound services as well as access to ultrasound services have been documented to differ by social class. A study in Nepal found that the adjusted odds of receiving an ultrasound exam were 3.4 times higher for women who had 7 to 10 years of education and 10.28 times higher for women who had more than 10 years of education compared to women with no formal education [42]. In sub-Saharan Africa, access to obstetric ultrasound in rural areas has shown to be as low as 6% [7, 31].

Providers’ explanations or lack of explanations about the ultrasound scan play a role in terms of patient perception and satisfaction. Women who received ultrasound services in a rural setting in Botswana described that turning the light off before the examination was an unusual experience [2]. Patients normally expect examination rooms to be “brightly lit” (p. 694) [2]. In a study in southeast Nigeria, 70% of study participants reported that they did not interact with the sonographers, 24% reported being afraid prior to the scan, 11% reported being afraid during the scan and 4% reported being afraid after the scan [51].

### What are potential issues with the ultrasound device?

#### Maintenance

Although there are no previous studies rigorously testing the durability of ultrasound machines in LMICs, some studies provide anecdotal accounts. Kozuki et al. describes having to replace refurbished portable ultrasound machines four times in the span of 17 weeks in a study in Nepal due to hardware and software errors [5, 52]. In another study by Kimberly et al., portable ultrasound machines performed well even in excess heat, humidity, dust and long travels in a 6-month study [6]. At the 1-year follow-up visit, however, 38% of the midwives who responded to a follow-up survey cited problems with the ultrasound machine [6]. These problems included depletion of ultrasound gel and flickering image on the ultrasound screen [6]. Another study reported that maintenance was not an issue during the 5-month study period [14]. In fact, a study conducted in extreme conditions of the Amazon jungle reported that portable ultrasound machines functioned well for two two-week trips [53]. However, there is limited evidence for maintenance beyond the research study period. One study describes that establishing a maintenance protocol within routine health systems can be long and difficult [24]. In this study, such difficulty left many ultrasound machines unrepaired [24].

#### Cost

In high-income countries, there has been many studies assessing the cost effectiveness of using ultrasound to identify fetal abnormalities, as demonstrated by a 2002 review [54]. Cost effectiveness studies are less common for LMICs. Only a few studies provided an overview of program costs and the realized benefits. Bussmann and colleagues reported that in a district hospital in Botswana, initial capital and recurrent costs of the ultrasound were considered affordable, relative to the overall unspent hospital budget in the study period [4]. They also found that “the marginal cost of providing an ultrasound was less than a quarter of providing an X-ray examination” (p. 1030) [4]. The average and marginal costs of an ultrasound diagnosis in improving patient management appeared to be affordable as well, 56.58 USD and 0.64 USD respectively [4]. Lastly, the average and marginal costs of potentially improving a health outcome through an actual change in therapy, were considered affordable, 260.79 USD and 2.93 USD respectively [4]. Overall, the study concluded that providing ultrasound scans in Botswana was financially feasible [4]. Another study in Nepal estimated the impact of ultrasound on the potential to avert perinatal deaths attributable to non-cephalic birth, multiple birth and placenta previa [5, 52]. The study estimated that with an early ultrasound diagnosis, a total of 160 potential perinatal deaths could be averted, which translates to about 65 USD saved per life [5, 52]. This estimation was derived based on the assumption that all early diagnoses will lead to the prevention of perinatal deaths [5, 52], likely overestimating the true impact. However, it also did not adjust for potential disability life years averted by preventing maternal mortality or morbidity [5, 52]. Hence, the estimated impact on averting potential perinatal deaths does not capture the full range of benefits that early ultrasound diagnosis offers.

In summary, there is a lack of high quality cost effectiveness studies on obstetric ultrasound in the literature [54]. The majority of published studies are from high-income countries, and they did not include discussions about longer term costs or cost incurred to women [54].

## Discussion

This review examined various factors associated with introducing obstetric ultrasound in LMICs. A recent cluster-randomized trial found that use of ultrasound in rural health centers did not impact antenatal care attendance, facility delivery, maternal mortality, neonatal mortality and stillbirths [20]. A related case study further suggested that scale-up of routine antenatal ultrasound is not warranted [55]. Other studies with less rigorous designs, however, found that ultrasound use was associated with the increase of antenatal care attendance [23, 25] and facility delivery [6, 7, 23, 25, 49]. Although antenatal ultrasound use did not affect mortality measures, there is evidence suggesting that ultrasound can confirm and improve patient management for both obstetric and non-obstetric conditions [4, 6, 8, 10–12, 14, 27, 28] . One potential concern with the introduction of ultrasound is whether it will disrupt existing services routinely provided at the health facility. Muller-Rockstroh describes that select district hospitals in Tanzania decided to train nurse-midwives for ultrasound use instead of training radiographers or radiography assistants [24]. This is because use of ultrasound by radiographers would have disrupted the X-ray services also provided by them [24]. Even when disruption of other services is not an issue, there is still a disagreement in the literature about routine versus selective provision of ultrasound services. Kongnyuy and van den Broek argue that ultrasonography should be routinely performed for all women because it is regarded as safe and affordable [1]. It could also save costs by detecting abnormalities early in pregnancy [1, 9]. Papp and Fekete support routine ultrasound screening on the basis that 85 to 90% of congenital malformations occur without maternal or family antecedents and therefore, selective ultrasound screening may miss a lot of cases that cannot be deducted based on previous medical history [56]. Tautz and colleagues offer a counter-argument, however, that there is insufficient evidence for ultrasound to be recommended as a routine screening tool [2]. Instead, they argue that selective use of ultrasound during the antenatal period can complement diagnoses that remain uncertain after other clinical tests have been performed [2]. Hofmeyr also adds that routine ultrasound services for patients with already confirmed pathologies may risk wasting human resources that could be allocated more efficiently elsewhere [9, 57].

While there is a lack of consensus in the literature, the WHO issued a brief which recommends that pregnant women receive only one ultrasound scan before 24 weeks gestation for accurate determination of gestational age, early identification of fetal anomalies and multiple pregnancies, appropriate preparation and management of preterm and post-term births and helping create a positive pregnancy experience [58]. For those who did not receive a scan during this period, a later scan may be considered to determine fetal number, presentation and placental location [58]. However, ultrasound may be used more than once depending on the specific patient condition.

Additional guidelines and considerations are discussed in the literature for large-scale implementation of ultrasound.

### Health personnel training

Mid- and lower-level health providers demonstrated competence in using ultrasound and in making accurate diagnosis with only a short intensive training [5, 6, 8, 10, 14, 17, 27, 29, 32–34]. Although past training programs were largely successful, a few points have been consistently highlighted in the literature for improving future endeavors.

First, because past training programs varied greatly in duration and content, there is a need for an internationally recognized standard of training or a certificate program [59, 60]. In addition, an ideal training module would minimize the interruption to local health workers’ schedules and allow for practical hands-on sessions and applications [59, 61].

Second, training programs should incorporate management lessons such as short-term and long-term maintenance of the ultrasound machine, image storage, image review and quality assurance as a part of the curriculum [18, 59]. Attention to these management topics will increase the likelihood that ultrasound machines are continuously being used after the training has concluded, particularly in rural and isolated locations [18, 59].

Third, training programs should ensure that continuing education is available for participants after the short intensive training courses conclude [59]. This may be in the form of follow-up refresher sessions, direct supervision, review of donated textbooks, review of publicly accessible or affordable journals, presentations and teleconferencing [3]. Remote learning and supervision via teleconferencing could potentially reduce costs and have a positive impact on the quality of care [62]. Providers in LMICs have also shown receptiveness to the possibility of distance learning modules [7, 60]. This option is especially appealing for rural health workers because commitment to lengthy training programs and continuous in-person follow up sessions may be unrealistic [61].

Fourth, training programs should sensitize health providers in legal and ethical conduct regarding ultrasound use and towards patients’ desire for being informed about their care [2]. This is because overuse and misuse [2, 37–39], determining fetal sex [1, 5, 9, 41] and the lack of communication between health providers and patients were found to be major concerns regarding ultrasound use in LMICs [2].

In the long term, a more sustainable solution would be to involve local radiology societies and training institutions to increase support for locally trained specialized sonographers [7, 63]. This trend is already underway as a number of training institutions and associations have been established in Africa in recent years [7].

### Ultrasound acquisition and maintenance

Several factors must be considered when acquiring ultrasound machines and setting up maintenance protocols [59, 64]. First, various features of the ultrasound machine – image quality, level of radiation and safety, easy and robust operability, transducers, ultrasound gel, supplies for cleaning, maintenance service and storage must be thoroughly considered [59, 64]. Second, and of key importance is the selection of an ultrasound machine that serves the purpose for which it is intended (e.g. the quality of the image resolution determines what can be diagnosed with a particular machine). Third, there must be a comprehensive assessment of the practice environment in which ultrasound will be used [64]. The reliability of electricity supply, the volume of patients received at the health facility, and the intended use/types of diagnoses expected may determine the local preference for full-size versus portable ultrasound machines [59]. For example, a high volume hospital with a more reliable source of electricity might prefer a full-size, plug-in ultrasound because of the larger size rendering protection against theft. For smaller health clinics operating in rural areas, using a battery-operated portable ultrasound machine may be more practical. Even with portable ultrasound machines, security measures can be taken to guard against theft or damage. These measures include storing the machines in a locked room or placing it under constant observation when there is a high demand for use [55, 59]. For health facilities that already own full-size ultrasound machines, however, purchasing portable ultrasound machines might be redundant [3]. Third, durability of ultrasound machines as well as the manufacturer’s local capacity for maintenance should be considered. It may also be important to train in-house mechanics to take primary responsibility over minor repairs [59].

### Cost-effectiveness

Recent studies that included descriptions about their program’s costs and the realized benefits concluded that providing ultrasound services was financially feasible and cost-effective [4, 5]. Yet, there is still a lack of high quality cost-effectiveness studies conducted in LMICs [54]. This calls for more studies to be conducted with rigorous designs [54], so that a true comparison of the added value of the service and the intervention costs versus the counterfactual can be made. It is also essential to define the package of services in which effectiveness is evaluated. Key questions to consider are how recommended antenatal ultrasound services [58] would be integrated into existing maternity services most effectively and what potential trade-offs exist with other essential services in resource-constrained settings. A recent case study from the Democratic Republic of the Congo reported that the success of implementing structural changes in the health system would rely on the level of stakeholder effort, motivation, political will and financial and human resources available [55]. In the absence of these elements, streamlined integration into the health system would be challenging.

### Promoting cultural competence

Although there was a tendency of overestimating the diagnostic capabilities of ultrasound [2, 7], patients generally seemed to hold positive views about ultrasound services [23]. Cultural resistance to ultrasound was also not a major issue [6, 49]. In certain cultures, however, viewing the insides of a pregnant mother or projecting the image of a fetus might be considered offensive [3]. Introduction of ultrasound services in these settings may require investment into community engagement about the benefits of ultrasound during pregnancy [3]. As one strategy, education efforts could highlight areas where conventional methods are similar to the procedures of ultrasound, so that any uneasiness related to trying new technology may be mitigated. In Tanzania, for example, the resemblance between applying ultrasound gel and rubbing local medicine on the women’s belly for diagnosing problematic pregnancies seemed to help establish women’s trust [24]. Including husbands in the intervention may also be a culturally appropriate strategy in some cases as one study found that they played a role in encouraging women to seek antenatal ultrasound exams [23].

## Conclusion

This literature review focused on obstetric uses of ultrasound in LMICs. As cost of obstetric ultrasound becomes more affordable in LMICs, it is essential to assess the benefits, trade-offs and potential drawbacks of large-scale implementation. Additionally, there is a need to more clearly identify the capabilities and the limitations of ultrasound, particularly within the context of limited training of providers, to ensure that the purpose for which an ultrasound is intended is actually feasible (e.g. the image quality of portable ultrasound is not sufficient to identify fetal anomalies and early gestational age). We found evidence of obstetric uses of ultrasound improving patient management. However, there was evidence that ultrasound use is not associated with reducing maternal, perinatal or neonatal mortality. Patients in various studies reported to have both positive and negative perceptions and experiences related to ultrasound and lastly, illegal use of ultrasound for determining fetal sex was raised as a concern.
